# Directed Signaling Cascades in Monodisperse Artificial Eukaryotic Cells

**DOI:** 10.1021/acsnano.1c04219

**Published:** 2021-09-27

**Authors:** Sunidhi C. Shetty, Naresh Yandrapalli, Kerstin Pinkwart, Dorothee Krafft, Tanja Vidakovic-Koch, Ivan Ivanov, Tom Robinson

**Affiliations:** † Theory and Bio-Systems, Max Planck Institute of Colloids and Interfaces, Am Mühlenberg 1, 14476 Potsdam, Germany; ¶ Max Planck Institute for Dynamics of Complex Technical Systems, Sandtorstrasse 1, 39106 Magdeburg, Germany

**Keywords:** microfluidics, enzyme cascade, bottom-up, synthetic biology, multicompartmentalization, directed signaling, artificial cells

## Abstract

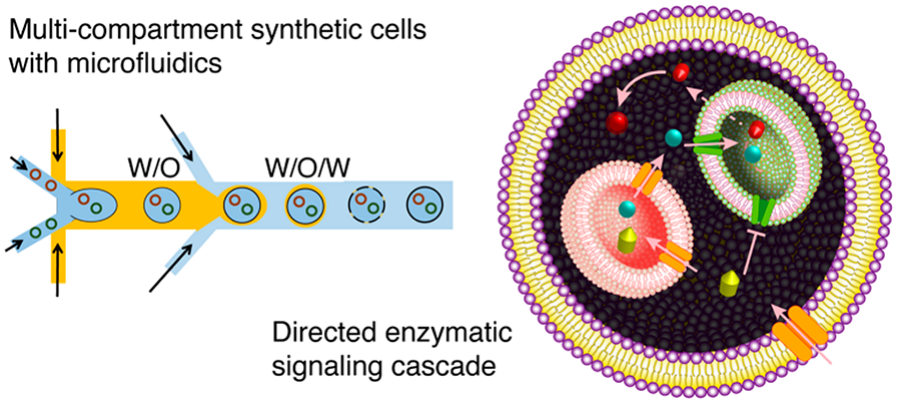

The bottom-up assembly of multicompartment artificial cells that are able to direct biochemical reactions along a specific spatial pathway remains a considerable engineering challenge. In this work, we address this with a microfluidic platform that is able to produce monodisperse multivesicular vesicles (MVVs) to serve as synthetic eukaryotic cells. Using a two-inlet polydimethylsiloxane channel design to co-encapsulate different populations of liposomes we are able to produce lipid-based MVVs in a high-throughput manner and with three separate inner compartments, each containing a different enzyme: α-glucosidase, glucose oxidase, and horseradish peroxidase. We demonstrate the ability of these MVVs to carry out directed chemical communication between the compartments *via* the reconstitution of size-selective membrane pores. Therefore, the signal transduction, which is triggered externally, follows a specific spatial pathway between the compartments. We use this platform to study the effects of enzyme cascade compartmentalization by direct analytical comparison between bulk, one-, two-, and three-compartment systems. This microfluidic strategy to construct complex hierarchical structures is not only suitable to study compartmentalization effects on biochemical reactions but is also applicable for developing advanced drug delivery systems as well as minimal cells in the field of bottom-up synthetic biology.

Intracellular compartmentalization is a key feature of eukaryotic cells, and they have evolved to exhibit a hierarchical architecture with spatiotemporal control over their metabolic processes.^1−3^ An essential prerequisite for complex metabolic reactions to occur was the evolution of membranous organelles, enabling directed and spatial segregation of biomolecules to perform complex reactions as well as to prevent deleterious subsidiary pathways.^4−6^ Owing to the complexity of pathways residing in eukaryotic cells, studying this effect *in vivo* is nontrivial and isolating individual pathways without the interference of others is impossible. Therefore, an emerging strategy is to reconstitute enzymatic pathways into biomimetic artificial systems mimicking both the cell itself and their organelles. This bottom-up approach has the advantage of near-complete control of the components and therefore allowing the study of mutual interactions of different pathways. Constructing complex multicompartment systems not only permits the exploration of enzymatic reaction cascades but is also key to developing artificial minimal cells in the context of bottom-up synthetic biology^7^ and for building advanced drug delivery platforms.^8^

Previously, a few complex multicompartment architectures based on liposomes,^9^ polymersomes,^10^ and polymer capsules^11^ have been reported. Perhaps the simplest approach to create multicompartment structures is to form multilamellar vesicles exhibiting onion-like structures by spontaneous hydration,^12^ but this method has poor control over their size and lamellarity. A more commonly used method to generate multicompartmentalized systems is the inverted emulsion method. Here water-in-oil (W/O) droplets containing preformed subcompartments such as proteoliposomes,^13,14^ multiple partitioning droplets,^15^ or intermediate-sized giant unilamellar vesicles (GUVs)^16^ are driven through a water–oil interface to create the outer membrane. While this method does offer separate control over the inner and outer aqueous compartments and their membranes, it can suffer from low or inhomogeneous encapsulation efficiencies. Alternatively, instead of encapsulating inner compartments within liposomes, a layer-by-layer technique can be employed to form multicompartment structures that mimic complex cellular architectures.^17^ However, this method results in a polydisperse size distribution and has limited space in between the compartments to perform reactions. Successful chemical communication has been demonstrated within multicompartment lipid-based structures by the permeation of trigger molecules^18^ and by using a temperature-controlled release of substrate molecules from the inner compartments.^19,20^ Although these examples have great potential for sensors, they possess limited control over their internal signaling directionality. Alternative approaches using polymersomes have shown promising triggered release strategies; however, these systems do not fully reflect biological membranes and display limited spatiotemporal control over the transport of molecules within them.^21,22^

These reported systems, which are produced either with inverted emulsions,^13−16^ electroformation,^18,23^ or other bulk methodologies, inherently have little control over size and reproducibility and possess low or varying amounts of inner compartments, all of which are important for high-throughput analysis of parallel biochemical reactions. In an attempt to address these issues, researchers are turning toward microfluidic methods.^24−30^ Droplet-based microfluidic approaches offer high-throughput formation, precise control over the size of the compartments, narrow size distributions, and small reaction volumes, making them ideal platforms to generate complex artificial cell models with precious cargos.^25^ Previously, a microfluidic-based approach was used to create multivesicular droplets, and a two-step diffusion-based enzymatic reaction was demonstrated.^26^ While droplet encapsulation of biological components does offer high encapsulation and size control, the lack of an outer lipid membrane prevents complex reconstitution of membrane-based cellular processes. Alternatively, living cells can be encapsulated within vesicles aided by droplet microfluidics to demonstrate enzymatic conversion.^27^ Such hybrid cells open up the possibility of using living cells as functional modules, but the reported method requires nonphysiological conditions with high sugar concentrations to drive the final bulk gravity-driven formation of the compartmentalized systems. Microfluidic layer-by-layer membrane assembly was also shown to successfully create controllable multilamellar structures with defined numbers of bilayers, but in the context of encapsulating reaction cascades, this approach would have limited control over the directionality.^28^ Glass capillary-based microfluidics can generate multivesicular vesicles (MVVs) with control over the number of inner compartments along with their encapsulated content and have been used to segregate protein synthesis in different compartments.^30^ While these microfluidic approaches show an advanced level of control, complex enzymatic cascades and directed pathways of intermediates have not yet been reported. Moreover, these above strategies have not been used to directly compare the effects of different hierarchical levels of compartmentalization on enzymatic pathways.

In this work, we employ a polydimethylsiloxane (PDMS)-based microfluidic platform for the easy fabrication and high-throughput formation of biomimetic MVVs. Monodisperse GUVs are created using a water-in-oil-in-water (W/O/W) double emulsion technique, and membranous compartments are formed by encapsulating large unilamellar vesicles (LUVs) in the inner aqueous (IA) solution. To avoid the fusion of the liposomes and loss of compartments, PEGylated lipids are employed in both the LUVs and in the lipid oil (LO) solution used for the production of double emulsion templates. We use these stable multicompartmentalized systems to construct signaling pathways mimicking those found in eukaryotic cells. A three-enzyme reaction pathway spanning across two different populations of inner LUVs was implemented. Co-encapsulation of the two liposomes was made possible by developing a two-inlet microfluidic design. Directed pathways of the reaction intermediates are achieved by reconstituting size-selective membrane pores at the boundaries of specific compartments. Finally, owing to the monodispersity of our MVVs, we are able to compare final product formation and overall reaction rates between bulk and confined systems with increasing order of complexity.

## Results and Discussion

### Microfluidic Formation of Monodisperse Multivesicular Vesicles

**Figure 1:**
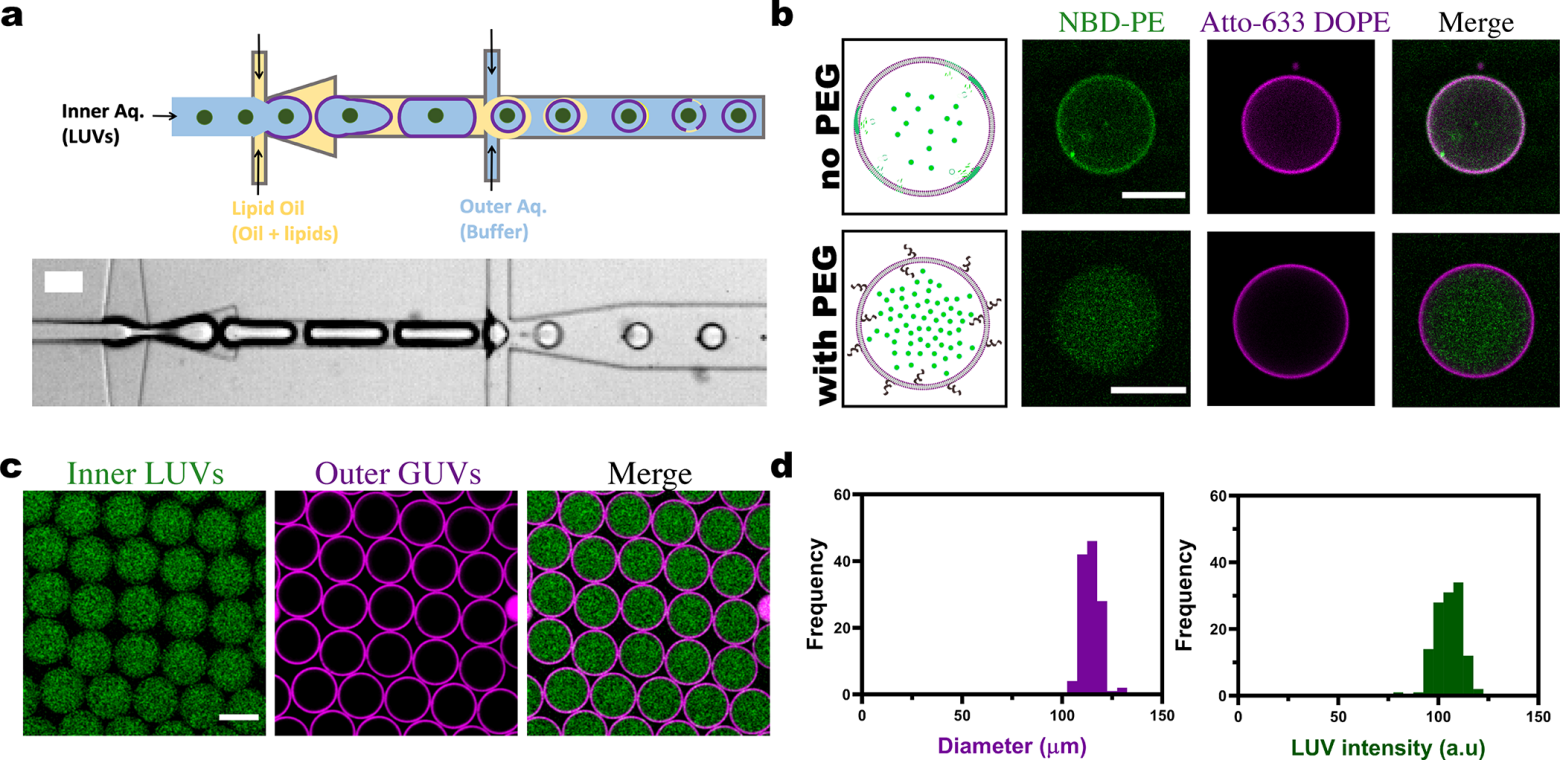
Microfluidic assembly of compartmentalized MVVs. (a) Schematic and bright-field image of the microfluidic platform. An aqueous phase containing LUVs forms water-in-oil droplets at the first cross junction, which are then sheared at the second cross junction to form double-emulsion templates. These undergo spontaneous dewetting of the oil to render MVVs. Scale bar: 100 μm. (b) Generation of MVVs in the absence of PEGylated lipids resulted in LUV bursting and aggregation at the inner leaflet of the GUVs (top). The addition of 1 mol % PEG-DSPE in the lipid mixture resulted in successful encapsulation of inner compartments (*i.e*., LUVs) in outer compartments (*i.e*., GUVs) without rupture (bottom). Inner LUVs consist of POPC:DOPG:Cholesterol:mPEG-DSPE with additional NBD-PE for labeling, and the outer POPC:DOPG:Cholesterol:mPEG-DSPE GUVs are labeled with Atto-633 DOPE. Scale bars: 50 μm. (c) Confocal fluorescence image of the monodisperse GUVs (purple) with inner LUVs (green). Scale bar: 100 μm. (d) Histograms showing the size distribution of MVVs and mean intensities of the inner LUVs (*n* = 123).

Figure 1 illustrates the construction of our MVVs generated with microfluidic double emulsion templating. In the first step, 400 nm diameter LUVs were formed using the lipid film hydration method followed by extrusion through polycarbonate membranes.^31^ These fluorescently labeled nanocompartments were then encapsulated within microfluidic GUVs formed using an octanol-assisted method to create compartmentalized systems.^32^ The IA containing LUVs is used to generate W/O droplets at the first cross junction, which are then sheared at the second cross junction to form W/O/W double emulsions (Figure 1a and Supplementary Movie S1). These serve as templates that undergo spontaneous dewetting of the oil phase to render GUVs and consequently the MVVs. To prevent unwanted LUV fusion and aggregation at the water–oil interface, additional PEGylated lipids were included in the membranes of our vesicles (both LUVs and GUVs). PEG-lipid conjugates are known to provide a steric barrier against the fusion and aggregation of LUVs.^33^ Here the use of PEGylated lipids enables both high encapsulation inside the microfluidic GUVs and avoids unwanted bursting or fusion of the LUVs to each other or with the host membrane (Figure 1b). The formed MVVs therefore remained stable and maintained their inner compartment integrity for the duration of the experiments (Supporting InformationFigure S1).

Figure 1c shows confocal images of the resulting MVVs formed with our microfluidic device. A high-throughput formation of MVVs with a uniform size distribution of 114.1 ± 4.7 μm with a relative standard deviation (RSD) of 4% was achieved (Figure 1d, left panel). Furthermore, homogeneous encapsulation of inner LUVs was attained with a uniform mean intensity of 104.9 ± 6.4 au and an RSD of 6% (Figure 1d, right panel). The absence of fluorescence signal (Figure 1c, green channel) at the membrane of GUVs shows no loss of inner compartments *via* LUV bursting. We also observed no fluorescence in the exterior of GUVs, further confirming successful high encapsulation of LUVs. This emphasizes the importance of employing PEGylated lipids in our system as well as the advantage of the microfluidic approach to generate highly robust and stable monodisperse artificial cells. The high-throughput formation of MVVs allows for faster analysis and testing of large populations of multicompartment systems of precise composition, and the monodispersity gives us the opportunity to later study the effects of confinement across increasingly complex compartments.

### Chemical Cascade Signaling in Microfluidic Artificial Cell Models

**Figure 2:**
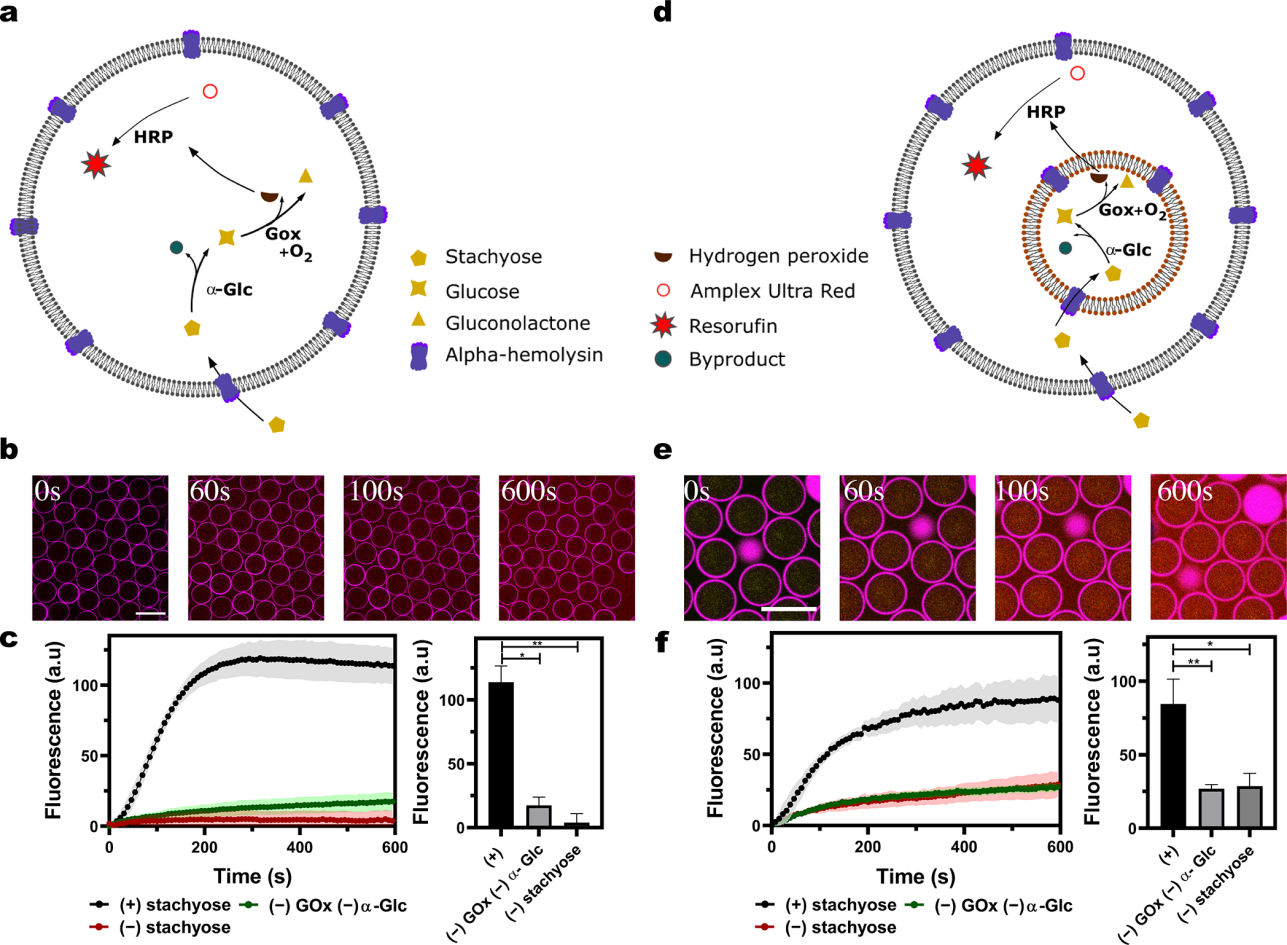
Chemical cascade reaction network in one- and two-compartment systems. (a) Scheme of a one-compartment system with HRP, GOx, and α-Glc in the lumen of GUVs functionalized with αHL pores. (b) Confocal fluorescence time series of multiple homogeneous one-compartment GUVs with a mean diameter of 75.3 ± 6.1 μm after being triggered externally with stachyose molecules *via* the αHL pores. (c) Average kinetic traces (left) and end point measurements (right) of the resorufin signal (*P* < 0.005, unpaired *t* test, *N* ≥ 2 for the one-compartment system, −stachyose and −GOx−α-Glc controls, respectively, *n* ≥ 50). (d) Schematic representation of the two-compartment system with HRP in the lumen of the outer GUV and with GOx and α-Glc further encapsulated within a population of LUVs (also embedded with αHL pores). (e) Confocal fluorescence time series of the MVVs with a mean diameter of 70.2 ± 4.9 μm after addition of stachyose. Note that the bright spots are the detached oil droplets. (f) Average kinetic traces (left) and end points (right) of the resorufin signal (*P* < 0.005, unpaired *t* test, *N* ≥ 2 for the two-compartment system, −stachyose and −GOx−α-Glc controls, respectively, *n* ≥ 50). Error bars in (c) and (f) are taken from the standard error of the mean. Scale bars: 100 μm.

The implemented synthetic signaling cascade is based around a three-enzyme reaction pathway (Figure 2a). First, the relatively large tetrasaccharide stachyose is broken down into smaller glucose molecules by α-glucosidase (α-Glc). In the presence of oxygen, glucose is then converted to gluconolactone and hydrogen peroxide (H_2_O_2_) by glucose oxidase (GOx). Finally, Amplex Ultra Red (AUR) is converted into fluorescent resorufin by horseradish peroxidase (HRP) in the presence of H_2_O_2_. Prior to incorporation into the MVVs, this complex reaction scheme was analyzed in bulk using a 96-well plate reader to establish optimal reaction conditions that would allow the efficient monitoring of the final fluorescence output. Parameters such as the enzyme concentrations (*i.e*., HRP, GOx, and α-Glc) and substrate concentration (*i*.*e*., AUR) were varied to achieve sufficient production of resorufin within a suitable timeframe so that the chemical cascade could be easily detected when confined in the MVVs. In particular, GOx-catalyzed conversion of glucose to gluconolactone and H_2_O_2_ was found to be the rate-limiting step, and therefore its concentration was adjusted accordingly (Supporting InformationFigure S2).

As a first step toward a synthetic signaling cascade in MVVs, the reaction network was encapsulated directly inside single-compartment GUVs, without LUVs, using the microfluidic device in Figure 1. For temporal control, the chemical trigger stachyose was excluded from the reaction mixture and introduced externally into the lumen of the GUVs by the functionalization of the membrane with alpha-hemolysin (αHL). This protein can self-assemble into lipid bilayers to form heptameric pores that enable the passage of small molecules such as stachyose, which would normally be membrane impermeable.^34^ To confirm the successful encapsulation of enzymes and to initiate the signaling cascade, the first step was to trigger the network externally with the addition of αHL pores (20 μg/mL). The three enzymes (*i*.*e*., α-Glc, GOx, and HRP) rapidly converted the fluorogenic substrate AUR into fluorescent resorufin signal within the GUVs (Figure 2a), which was measured by confocal fluorescence microscopy (Figure 2b). Multiple GUVs were monitored simultaneously from the time point of initiation and for a period of 10 min, within which the signal plateaued. The increase in signal after triggering with stachyose (Figure 2c, left panel, black line) was higher compared to that measured in the absence of stachyose (red line) as well as in the absence of the enzymes GOx and α-Glc (green line). End point measurements revealed that the average fluorescence signal after 10 min was significantly higher than the controls without stachyose or GOx and α-Glc (Figure 2c, right panel). Due to the homogeneous size distribution of the formed GUVs, we observe very low error and high reproducibility in the product formation levels and reaction times.

Having demonstrated the ability to externally trigger the three-enzyme coupled reaction network inside single-compartment GUVs, we then aimed at increasing the spatial control by further confining specific enzymes within two distinct compartments in MVVs. This was achieved by encapsulating HRP alongside a population of LUVs containing both GOx and α-Glc (see Methods) in microfluidic GUVs using the setup described in Figure 1. In order to externally trigger the entire reaction cascade, αHL was incorporated into the membranes of MVVs, allowing the stachyose molecules to enter the αHL-incorporated inner LUVs and subsequently trigger the formation of the final product, resorufin, within the lumen of the GUV (Figure 2d). The change in resorufin intensities within the MVVs is shown as a confocal time series in Figure 2e. It should be noted that the successful functionalization of the membranes with αHL also confirms the unilamellarity of our GUVs. The resorufin fluorescence was measured for each GUV after the cascade reaction was triggered with stachyose molecules (Figure 2f, left panel, black line) in contrast to control experiments, in the absence of stachyose (red line) as well as in the absence of GOx and α-Glc (green line). End point measurements revealed a significant increase in resorufin fluorescence in comparison with the controls (Figure 2f, right panel). Moreover, the inner LUVs were able to maintain their integrity throughout the reaction duration while containing enzymes and being functionalized with membrane proteins (Supporting InformationFigure S3).

We observe that the overall rate of product formation for the two-compartment system is slower than the one-compartment system with the same concentration of the enzymes and substrate molecules per GUV. This could be attributed to the enhanced diffusion resistance that the input and/or intermediate molecules experience due to the additional membrane barriers of the inner LUVs, *i*.*e*., in the case of a two-compartment system. Moreover, the availability of the second substrate of GOx, namely oxygen, is also lower in the two-compartment compared to the one-compartment system (due to the higher diffusion resistance for oxygen). For both systems the intermediates can permeate out. However, in the case of LUVs this effect will be more pronounced compared to GUVs due to a higher surface-to-volume ratio. Consequently, the availability of a glucose substrate (one of intermediates) for the follow-up reaction with GOx could be lower in the LUVs compared to GUVs. It should also be noted that the local concentrations of enzymes are higher inside the LUVs for the two-compartment system (in order to keep the enzyme concentrations per GUV the same), which causes a higher glucose concentration gradient and efflux out of the LUVs.

**Figure 3:**
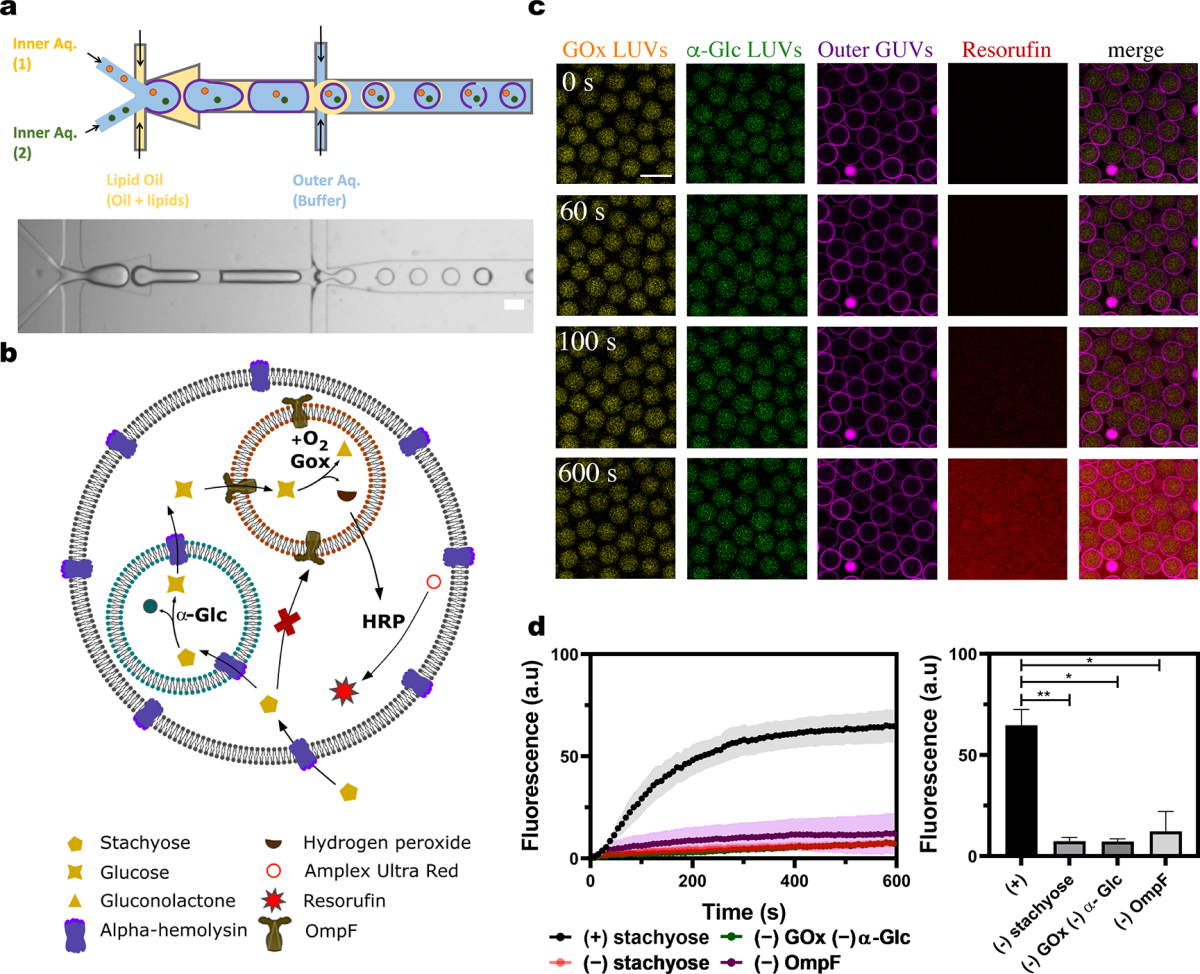
Chemical cascade reaction network in three-compartment MVVs. (a) Scheme and bright-field image of the two-inlet platform encapsulating two distinct LUV populations in the double-emulsion templates and subsequently undergoing dewetting to render MVVs. (b) Scheme of three-compartment MVVs with an outer GUV enclosing GOx-LUVs, α-Glc-LUVs, and HRP. (c) Confocal fluorescence time series of the MVVs with a mean diameter of 73.1 ± 7.2 μm after input of the chemical trigger. Channel 1 shows GOx containing LUVs tagged with Atto-390-DOPE and reconstituted with OmpF pores. Channel 2 shows α-Glc containing LUVs tagged with NBD-PE and embedded with αHL pores. Channel 3 shows the outer microfluidic GUVs tagged with Atto-633-DOPE, and channel 4 shows the resulting resorufin fluorescence over time. (d) Average kinetic traces (left) and end points (right) of the fluorescent resorufin product formed as a final output of the successful initiation of the enzyme cascade (*P* < 0.005, unpaired *t* test, *N* ≥ 2 for the whole system, −stachyose, −OmpF, and −GOx−α-Glc controls, respectively, *n* ≥ 50). Error bars are taken from the standard error of the mean. Scale bars: 100 μm.

The above demonstrates the ability of our MVV constructs to serve as externally triggerable and reproducible microreactors with spatially separated compartments. The final goal, however, was to create more complex MVVs architectures with three distinct compartments by co-encapsulating two different populations of LUVs within the microfluidic GUVs (Figure 3a). One population contains GOx, while the other contains α-Glc, and HRP is directly encapsulated within the lumen of the GUVs. Moreover, we aimed to direct the cascade reaction in a specific sequence of compartments, thus further advancing the spatial control of our MVVs. This contrasts with previous works, which rely on the homogeneous diffusion of the intermediates to all the compartments simultaneously.^26,35^ Therefore, we implemented a strategy based on size-selective membrane channels to direct the reaction intermediates to specific compartments sequentially. This was achieved by introducing a second membrane protein, enabling different transport properties of two distinct sets of LUVs. Stachyose, which has a molecular mass of 666.66 Da, can only enter the LUVs containing α-Glc (α-Glc-LUVs) *via* αHL pores. The resulting glucose with a molecular mass of 180.15 Da can then pass into the LUVs containing GOx (GOx-LUVs) *via* OmpF pores, which prevents molecules above 400 Da from entering (Figure 3b).^36^ This forms a basis to explore the size-selective chemical communication between these synthetic organelles (inner LUVs) within our artificial cells (outer GUVs).

After these two LUV populations with different membrane and internal compositions are formed (see Methods) they were encapsulated inside GUVs using a microfluidic platform with two inlets (Supplementary Movie S2). This device keeps the two populations of LUVs separate before encapsulating them into the W/O droplets formed at the first junction. This separation is partly necessitated to avoid cross-contamination of the LUVs with leftover membrane pores (if any), but it also promotes efficient co-encapsulation of the two inner LUV populations and forms distinctive subcompartments yielding more complex MVVs. This design could be of further interest for future applications that necessitate the components to be kept segregated before encapsulation. As before, temporal control is maintained by the external addition of stachyose trigger molecules that enter the MVVs *via* αHL in the outer GUV membranes. Subsequently, stachyose can only enter the α-Glc-LUVs where it is broken down into smaller glucose molecules, which in turn diffuse into the GUV lumen. Glucose can then enter the GOx-LUVs, where it is converted to gluconolactone and H_2_O_2_. H_2_O_2_ freely permeates across the LUV membranes into the GUV lumen, where the enzyme HRP converts AUR into fluorescent resorufin. Confocal fluorescence images show the buildup of this final product in the MVVs after the addition of the external trigger (Figure 3c). The co-encapsulation of both LUVs was uniform, and no bursting of LUVs during encapsulation was observed (Supporting InformationFigure S4 and Movie S3). Figure 3d shows that the final product resorufin was only observed in the presence of the trigger stachyose (black line), and the control without stachyose (red line) showed little increase in signal when averaged over all experiments. Moreover, a control without α-Glc and GOx (green line) showed only a slight increase in the final resorufin intensity. An additional control was performed in the absence of OmpF in the GOx-LUVs (violet line), and the negative result further demonstrates that the LUV population is maintained, as the membrane must be made permeable for the reaction to proceed. End point analysis revealed a significant increase in resorufin fluorescence in comparison with the four controls performed (Figure 3d and Supplementary Movie S4). Taken together, the observations described above confirmed the activation of the enzyme cascade within three-compartment MVVs and show the directed chemical communication between the synthetic organelles *via* size-selective membrane pores within our artificial eukaryotic cells.

### Compartmentalization Effects

**Figure 4:**
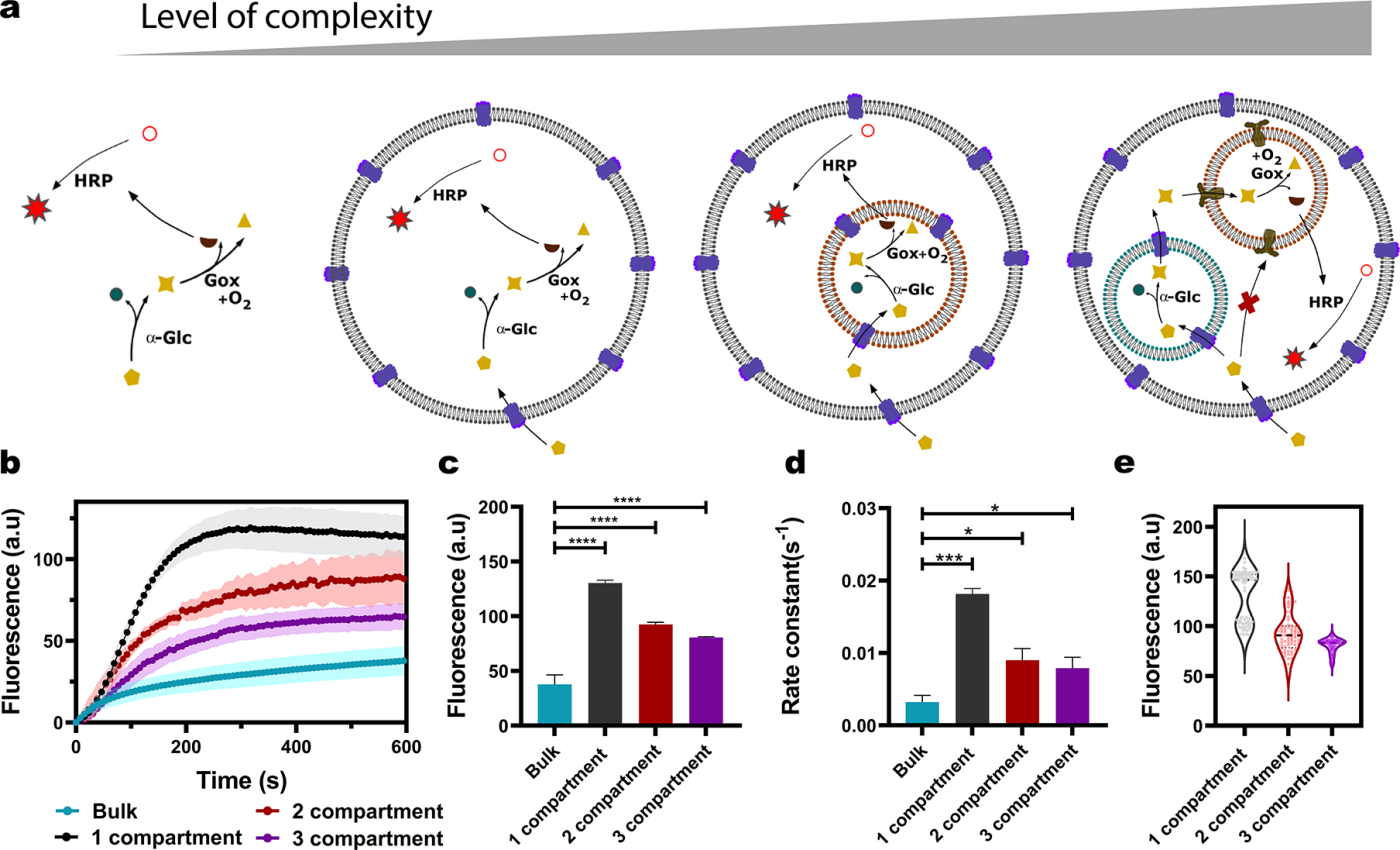
Comparison of the three-step enzyme reaction cascade network in systems with increasing complexity. (a) Schematic representation of the increasing levels of complexity from the open system to multicompartmentalized systems. (b) Average kinetic traces of bulk and one-, two-, and three-compartment systems. (c, d) Final resorufin intensities and apparent rate constants respectively (*N* ≥ 2). Error bars in (b)–(d) are taken from the standard error of the mean. (*P* < 0.05, unpaired *t* test, *n* = 6, 85, 88, and 116 for the bulk system and single-compartment, two-compartment, and three-compartment systems, respectively). (e) Violin plots of resorufin intensities across each of the different compartmentalized systems.

To better understand the role of compartmentalization in our systems, we compared the functionality of microfluidic-based one-, two-, and three-compartment MVV systems with an open bulk system (when the enzyme reaction network was not confined) (Figure 4a). We are able to make direct comparisons across all four of these systems of increasing complexity for the following reasons: (1) the enzyme and AUR concentrations per MVVs are carefully adjusted to be comparable across them all, (2) the input concentration of the trigger molecule is the same, and (3) the concentrations of enzymes and substrates are equal across the microfluidic GUVs owing to their high monodispersity and uniformity.

We observed that compartmentalization influenced the kinetics of the final product in the reaction cascade (Figure 4b). With the same concentration of trigger molecules and fluorescence substrates, the steady-state rate of the final product formed within the confined systems was higher than the open one. The end point analysis of the MVVs validated this observation; the resorufin fluorescence intensities measured in the compartmentalized systems were significantly higher than the open system (Figure 4c). It has been reported that membrane surfaces, as well as confinement, have a significant effect on the overall kinetics of enzyme activity.^37,38^ However, it is likely that the observed effect is due to a greater diffusion length for the reactant/intermediate transfer (to be able to reach their reaction sites) in the open system compared to the compartment systems. This is confirmed by the higher overall reaction rate constants for one, two, and three compartments compared to the open system (Figure 4d). Note that the enzyme concentration per GUV, where the data are extracted and analyzed from, is comparable (see Methods section). The observed rate constants, which are lower in two and three compartments compared to the one-compartment system, are most likely due to the additional membrane barriers slowing down the transport of input and intermediate molecules to the reaction sites of the enzymes (especially for the three-compartment system). This has a major contribution toward the variations in the final product formed, as seen in the end point steady-state analysis of one-, two-, and three-compartment systems. Violin plots revealed the relative distributions of the final product intensities across each of the compartmentalized systems (Figure 4e). A bimodal distribution of final intensity data in a single-compartment system implies a higher variance in the reaction product yield compared to the two- and three-compartment systems. A narrower normal distribution of intensities observed in the three-compartment system can be understood as an increase in the regulation of biochemical processes when the enzymes are spatially separated within distinct compartments. Segregation of the three enzymes could potentially lead to a reduction of any cross-enzyme inhibition. Moreover, it is expected that the enzyme–substrate equilibrium in a compartmentalized system is shifted more toward the direction of enzyme–substrate complex formation as the product diffuses out of the compartment and into the next one, which leads to a higher overall rate of reaction.^39^ Both of these effects reduce the probability of low product output. Furthermore, due to a greater number of membrane barriers present within a three-compartment system, the formed intermediates have a greater chance of leaking out of the system rather than forming a resorufin product and thereby reducing the probability of high product output. A combination of both these effects could explain the reduced product variability and the resulting regulation observed in the three-compartment system. However, a more detailed investigation is required to understand how the varying degrees of complexities in compartmentalization itself play a role in the kinetics of the enzymatic reaction cascade.

## Conclusions

We have described the generation of biomimetic MVVs comprising a directed three-step enzyme signaling cascade spanning across two inner compartments (LUVs) encapsulated within an outer compartment (microfluidic GUV) that can be triggered externally. First, we introduced a one-inlet microfluidic design to generate large populations of compartmentalized systems, thereby having control over the formation and loading of the compartments. The biocompatible multicompartment systems are modular and can be used as a chassis to mimic hierarchical architectures found in eukaryotic cells. Here, we highlight the addition of PEGylated lipids, as it prevented the bursting of inner LUVs and loss of material, thereby maintaining homogeneous encapsulation in the GUVs, which is further explored to build more complex and robust systems.

Second, we successfully developed a two-inlet design to achieve complex hierarchical multicompartmentalized structures. Size-selective behavior was realized with the incorporation of αHL (larger pores) and OmpF (smaller pores) in the two inner LUVs and comprising three different enzymes (*i*.*e*., HRP, GOx, and α-Glc) in three distinct compartments, therefore achieving directed spatial control. Moreover, the two-inlet microfluidic system has the advantage where one could vary the flow rates to obtain different ratios of encapsulated inner compartments within the MVVs for future applications. In this work, we chose to fabricate our microfluidic device from PDMS rather than glass capillaries, which have also been used to form multicompartment structures. While PDMS-based microfluidic platforms do possess limited solvent compatibility, they are easier to fabricate and operate compared to glass capillaries, which require complex alignment procedures.^40^

We note that the size of the formed MVV has an influence over the final output of the encapsulated cascade reaction. It was observed that larger MVVs had higher fluorescence intensities in comparison with the smaller MVVs (Supporting Information, Figure S5). This can be attributed to the fact that smaller MVVs have a greater surface-to-volume ratio and therefore have a higher probability of leakage of the intermediates compared to larger MVVs for a given reaction. This was confirmed with a diffusion experiment on two populations of GUVs with varying size diameter to determine the resorufin leakage kinetics (see Methods section and Supporting Information, Figure S6). We ascertained a higher probability of leakage of intermediates in smaller MVVs in comparison with larger MVVs, which results in higher product intensities in larger compartmentalized systems. Therefore, in our study, we were able to carry out a comparative analysis across all our systems because we were able to maintain a uniform size with the help of microfluidic approaches. In future applications, this could be taken advantage of, in order to tune the rate of reactions in compartments. In our system, the membrane compartments segregate the enzymes into specific locations, but with the different permeabilities due to OmpF and αHL pores, the intermediates and input molecules are also separated within the system to increase the efficiency and reduce interference from competing reactions. This reflects eukaryotic cell compartments, which is essential for building even more complex structures in the future. In this current setup, the intermediates and input molecules are spatially separated depending on the size-selective bias rendered by the pores reconstituted into the inner and outer compartments. While this provides directionality to the reaction network, it also limits the system, as intermediates can also diffuse out. To mitigate this, active transporters could be incorporated into the MVV membranes to minimize the loss but still provide directionality. This would also provide an opportunity to have a wider range of biotechnological applications in the future. However, one advantage of our system is that although the formation of intermediates and products results in an increase in the internal osmotic pressure, the effect is minimized due to the presence of membrane pores.

To explore the effects of compartmentalization, we compared the reaction kinetics of an open bulk system to those in confined environments. The results show that compartmentalization influences the overall kinetics of the biochemical reaction given the same concentration of reactants calculated per GUV. An increase in the overall rate was observed in confined systems compared to bulk. In order to better understand the effect that confinement has on the enzyme cascade kinetics, theoretical models would need to be developed to understand the critical parameters affecting the turnover rate at each step of the signaling pathway. This would allow optimization of different parameters such as concentrations of different components and pore densities to obtain desired membrane flux. So far, modeling of reaction networks to predict leakage kinetics in multilamellar vesicles has been reported,^41^ although a more complete model that considers the varying permeabilities of the inner compartments in a multicompartment system remains to be developed. We also observed an increase in the regulation of the multistep reaction cascade in our three-compartment system. Future work will focus on exploring this effect further and to encapsulate other reaction networks.

We have used a bottom-up approach and microfluidics to construct an artificial cell that mimics the behavior of eukaryotic cells that are able to sense/uptake metabolites and carry out downstream signal processing in specific organelles.^42^ In the future, we see such highly controllable systems being used to study and understand more complex signal transduction mechanisms found in eukaryotic cells. Finally, these multicompartment structures could be further developed in the future for advanced drug delivery platforms,^8^ for well-defined microreactors, and in synthetic cell research.^43−46^

## Materials and Methods

### Materials

All phospholipids 1-palmitoyl-2-oleoyl-*sn*-glycero-3-phosphocholine (POPC), 1,2-dioleoyl-*sn*-glycero-3-phosphocholine (DOPC), 1,2-dioleoyl-*sn*-glycero-3-phospho-*rac*-(1-glycerol) sodium salt (DOPG),1,2-diphytanoyl-*sn*-glycero-3-phosphocholine (DPhPC), cholesterol (ovine wool, >98%) (Chol), and fluorescence-labeled 1,2- dioleoyl-*sn*-glycero-3-phosphoethanolamine-*N*-(7-nitro-2-1,3- benzoxadiazol-4-yl) (NBD-PE) were purchased from Avanti Polar Lipids. 1,2-Dioleoyl-*sn*-glycero-3-phosphoethanol-amine labeled with Atto 633 (Atto 633-DOPE), phosphate-buffered saline (PBS buffer), bovine serum albumin, stachyose, α-hemolysin, glucose oxidase, and α-glucosidase were purchased from Sigma-Aldrich. 1,2-Dioleoyl-*sn*-glycero-3-phosphoethanolamine labeled with Atto 390 (Atto 390-DOPE) was purchased from Atto-Tec. Amplex Ultra Red was ordered from ThermoFisher Scientific. PD-10 columns were purchased from GE Healthcare. Horseradish peroxidase was purchased from Serva. Protein LoBind Eppendorf tubes and Safe-Lock tubes were purchased from Eppendorf AG. Osmometer measuring vessels for an Osmomat 3000 were purchased from Gonotec. Polydimethylsiloxane and curing agent were obtained as SYLGARD184 silicone elastomer kit from Dow Corning. 1*H*,1*H*,2*H*,2*H*-Perfluorodecyltrichlorosilane was purchased from abcr GmbH. Poly(diallyldimethylammonium chloride (PDADMAC) and poly(sodium 4-styrenesulfonate (PSS) were obtained from Sigma-Aldrich. SU8 2050 and SU8 developer solution were from Microchem Inc. Silicon wafers were purchased from Siegert Wafers.

### Methods

#### Generation of GOx-LUVs and α-Glc-LUVs

LUVs were formed using the thin-film hydration and extrusion method. A lipid mixture (5 mg/mL) containing POPC:DOPG:Cholesterol:mPEG-DSPE:Atto-390 in a 78.9:10:10:1:0.1 ratio was dried in a 5 mL glass vial under argon and placed under vacuum for 2 h. The lipid film was then rehydrated with a 1× PBS buffer containing 2 U/mL α-Glc to a final concentration of 5 mM. It was then freeze–thawed three times followed by extrusion 11 times with a 400 nm filter. The lipid composition for GOx-encapsulating LUVs was POPC:DOPG:DPhPC:Cholesterol:mPEG-DSPE:NBD-PE in a 68.5:10:10:10:1:0.5 ratio and was prepared following the same procedure and rehydrated with 1× PBS buffer containing 8 U/mL GOx.

#### Reconstitution of OmpF in GOx-LUVs

The reconstitution was carried out as in a previously reported protocol.^47^ Purified stock OmpF (5.5 mg/mL) in a detergent, a 1% solution of *n*-octylpolyoxyethylene (octyl-POE, Bachem) prepared in Millipore water, was diluted 1:1 in the same detergent and vortexed. A 1 μL amount of this freshly diluted OmpF solution was added to 199 μL of the GOx-LUVs solution and incubated at room temperature for an hour. GOx-LUVs embedded with OmpF pores were flowed through PD-10 columns to remove the detergent and unencapsulated enzymes. The eluted volume containing LUVs was collected for further encapsulation.

#### Assembly of αHL in α-Glc-LUVs

α-Glc-LUVs were preincubated with a final concentration of 5 μg/mL αHL, which can self-assemble to form pores of 1.2 nm in diameter and only allows molecules less than 3 kDa to pass through.^48^ α-Glc-LUVs embedded with αHL pores were flowed through PD-10 columns as previously described and then collected for further encapsulation.

#### Microfabrication and Surface Treatment

Master molds of 4 inch silicon wafers were prepared using standard photolithographic techniques as described before.^49^ Prebaked (65 °C for 3 min and 95 °C for 9 min) wafers were spin-coated with SU8 2050 to a height of 80 μm (model no. WS-650MZ-23NPPB, Laurell Tech. Corp.). Film masks (Micro Lithography Services Ltd.) with single- and double-inlet designs were then used to pattern them *via* UV light exposure for 8 s, followed by a postbaking step (65 °C for 2 min and 95 °C for 7 min). After revealing the unpolymerized SU8 using developer solution (Microchem Inc.), a hard bake step (30 min at 200 °C) was performed. To make the microfluidic devices, the PDMS monomer was mixed with the curing agent at a ratio of 10:1 and poured onto the master molds, which were pretreated overnight with 50 μL of 1*H*,1*H*,2*H*,2*H*-perfluorodecyltrichlorosilane in a vacuum to prevent adhesion. Following a curing step (3 h at 90 °C), the PDMS was removed, diced, and bonded to freshly cleaned glass coverslips (600 mbar for 1 min, Plasma Cleaner PDC-002-CE, Harrick Plasma) after punching respective inlets and outlets using a 1 mm biopsy puncher (Kai Europe GmbH). The microfluidic devices are preheated for 2 h at 60 °C before performing the hydrophilic surface treatment of the outer channel.

To form a stable W/O/W double-emulsion template, PDMS and the bonded glass coverslip are surface treated to render them hydrophilic at the second cross junction (for both single- and double-inlet devices). This was achieved by flushing a series of fluids, HCl/H_2_O_2_ (1:2) for 30 s, 2 wt % PDADMAC for 2 min, and 5 wt % PSS for 2 min, with Milli-Q water for 30 s after every step, from the outlet to the outer aqueous inlet. This process yields a functional microfluidic device to be used immediately thereafter.

#### Microfluidic Generation of Compartmentalized Systems

Double-emulsion templating for the formation of microfluidic GUVs is conducted using the procedure described elsewhere.^50^ Briefly, either enzymes, LUVs, or both (in 1× PBS buffer) or enzyme solution as the IA was pumped through the microfluidic chip (using a pressure pump, MFCS-EZ, Fluigent Inc.) to be sheared into uniform-sized W/O droplets at the first junction using a lipid mix of 5 mg/mL concentration in 1-octanol. The W/O droplet suspension was further sheared by pumping PBS buffer as the OA at the second junction to form double-emulsion templates. These double-emulsion templates, after the spontaneous dewetting process, result in GUVs containing LUVs. In the case of the two-inlet microfluidic device, both inlets are supplied with two different LUVs in PBS buffer, plus enzymes only with α-Glc-LUVs, as the IA solutions for the formation of the three-compartmentalized system. For the one-compartmentalized system, the enzyme solution was used as the IA solution.

To generate two-compartment systems (Figure 1) with a mean diameter of 114.1 ± 4.7 μm, we used pressures of 70 mbar at the IA (inner aqueous solution containing LUVs), 96 mbar at the OA (outer aqueous solution), and 79 mbar at the LO. For one-compartment systems with a mean diameter 75.3 ± 6.1 μm, we used pressures of 64 mbar at the IA, 50 mbar at the OA, and 74 mbar at the LO, and for the two-compartment systems with a mean diameter of 70.2 ± 4.9 μm, we used pressures of 65 mbar at the IA, 110 mbar at the OA, and 95 mbar at the LO (Figure 2). For the three-compartment systems with a mean diameter 73.1 ± 7.2 μm, we used pressures of 90 mbar at the IA (inlet 1), 90 mbar at the IA (inlet 2), 65 mbar at the OA, and 115 mbar at the LO (Figure 3). The generated MVVs were then collected and imaged on a bovine serum albumin (BSA)-coated coverslip. The trigger molecule, stachyose, with a final concentration of 50 mM was added along with αHL (20 μg mL^–1^) and AUR (10 μM) to initiate the reaction cascade in one-, two-, and three-compartment systems.

#### Calculating the Enzyme Concentrations within the MVVs

To keep the enzyme concentrations identical within all the systems used in this study, the concentrations of the LUVs were calculated using a Triton X-100-based solubilization assay.^51^ Briefly, LUVs produced after extrusion and PD-10 column separation were solubilized using 1% Triton X-100 containing 1× PBS buffer. Using fluorescence spectroscopy measurements from a Cytation 5 well plate reader, peak intensities for Atto-390 and NBD labeled lipids containing Triton X-100 were compared to that of starting concentrations of 5 mM lipid solutions in PBS for lipid concentrations determination (Figure S7). The lipid concentrations were then used to calculate the concentration of LUVs in the MVVs. Finally, concentrations of the enzymes were calculated using the equation below:
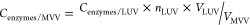
where *C*_enzymes/LUV_ is 1 μM for GOx and 1.7 μM for α-Glc and *n*_LUV_ is given by the number of LUVs enclosed in one GUV, which is defined by *n*_LUV_ = *C*_LUV_*V*_GUV_. Therefore, the calculated final enzyme concentration (*C*_enzyme/MVV_) for GOx was 5.1 nM and for α-Glc, 9.2 nM. The same enzyme concentrations were then used in the one-compartment systems and the bulk system.

#### Diffusion Experiment to Determine Leakage of Intermediates within GUVs

GUVs were prepared in a 300 mM sucrose solution using the electroformation^52^ method with the same lipid composition that was used for the microfluidic GUVs as described previously. The GUVs were introduced inside a microfluidic device containing traps to spatially confine them.^52^ This was done by applying a reverse flow using a syringe pump at a flow rate of 5 μL min^–1^. The outer solution was exchanged with 10 μM membrane permeable fluorescent resorufin at a flow rate of 0.2 μL min^–1^, and the influx kinetics of resorufin molecules were determined by recording a confocal time series. For the efflux, the resorufin solution surrounding the GUVs was exchanged with sucrose solution at a flow rate of 0.2 μL min^–1^. A single-exponential Boltzmann function *y* = A2 + (A1 – A2)/(1 + exp((*t* – τ)/d*t*)) was fit to the kinetic data shown in Figure S6 using OriginPro 9 to measure the half-time constant.

#### Microscopy and Quantitative Image Analysis

A Leica TCS SP8 confocal microscope equipped with an HC PL FLUOTAR L 20×/0.40 dry objective was used. Atto-633 was excited at 638 nm, and fluorescence was detected at 650–720 nm. Resorufin was excited at 552 nm and was detected at 583–616 nm. Excitation of NBD was performed with an excitation wavelength of 488 nm, and the signal was obtained at 499–542 nm. For excitation of Atto-390, the 405 nm excitation was used, and fluorescence was detected at 415–476 nm. Time series were recorded, and images were taken at 7.7 s per frame. A high-speed camera (MicroLab 310, Vision Research Inc.) fitted to an Olympus IX73 microscope was used to acquire bright-field images of the microfluidic droplet formation at ∼3000 fps. Images were analyzed using ImageJ FIJI and a custom-written code in Python. Apparent time constants are calculated by fitting the kinetic curves to a single-exponential function.

## Supporting Information Available

Movie S1: Microfluidic production of MVVs using a one-inlet device (AVI)
Movie S2: Microfluidic production of MVVs using a two-inlet device (AVI)
Movie S3: Time-lapse video of the three-compartment system containing two populations of LUVs (AVI)
Movie S4: Resorufin formation (red) in the three-compartment system (AVI)
Intensities and confocal images of encapsulated PEGylated LUVs; optimization of the three-enzyme reaction cascade in bulk; intensities and confocal images of inner LUVs in the two-compartment system; intensities and confocal images of GOx-LUVs and α-Glc-LUVs in the three-compartment system; effects of MVV size on product formation in the three-compartment system; lipid concentration calculated inside the LUVs; diffusion of resorufin into and out of GUVs (PDF)

## Supplementary Material

nn1c04219_si_001.avi

nn1c04219_si_002.avi

nn1c04219_si_003.avi

nn1c04219_si_004.avi
