# Supplementary material for: Directed Signaling Cascades in Monodisperse Artificial Eukaryotic Cells
Source: ACS Nano. 2021 Sep 27;15(10):15656–66. doi: 10.1021/acsnano.1c04219 (PMC8552445; doi:10.1021/acsnano.1c04219)
Supplement: Supplementary file 5 — nn1c04219_si_005.pdf [file nn1c04219_si_005.pdf]

Supplementary Information for

Directed Signaling Cascades in Monodisperse Artificial Eukaryotic Cells

Sunidhi C Shetty [†], *Naresh Yandrapalli* [†], *Kerstin Pinkwart* [†], *Dorothee Krafft* [¶], *Tanja Vidakovic-Koch*[¶], *Ivan Ivanov*[¶], *Tom Robinson* [*,†]

[†]Theory and Bio-Systems, Max Planck Institute of Colloids and Interfaces, Am Mühlenberg 1, 14476 Potsdam, Germany

[¶]Max Planck Institute for Dynamics of Complex Technical Systems, Sandtorstrasse 1, 39106 Magdeburg, Germany

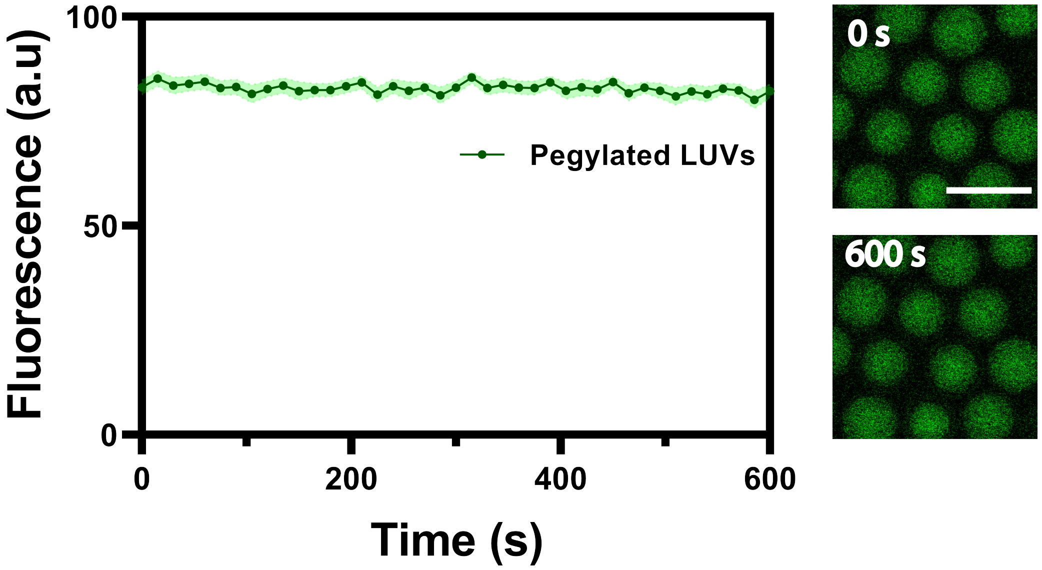

Figure S1. Intensities of encapsulated PEGylated LUVs fluorescently labeled with NBD-PE within the two-compartment system, together with confocal images at 0 and 600 s. Error bars are taken from the standard error of the mean (n = 53). Scale bar: 100 μm.

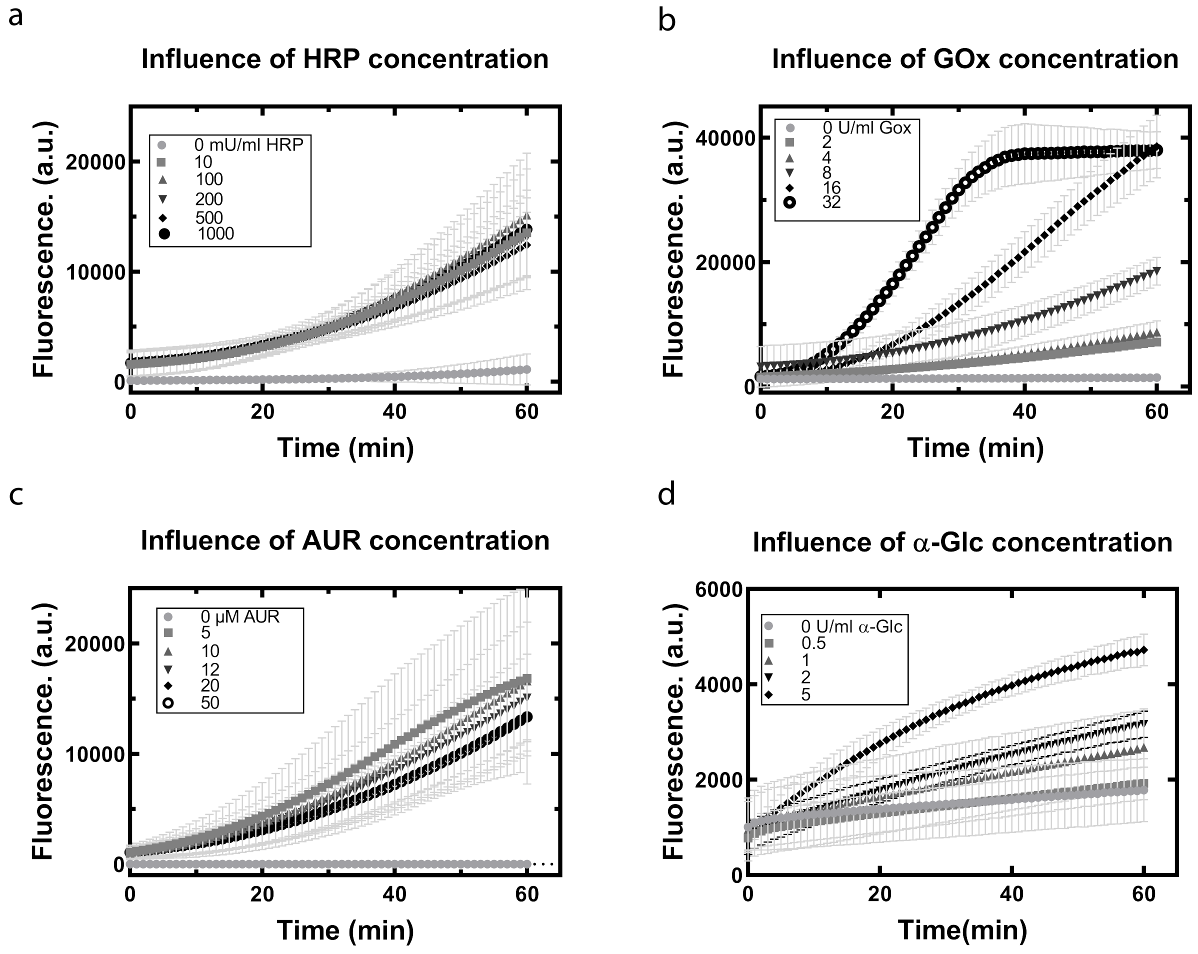

Figure S2. Optimization of the three-enzyme reaction cascade in bulk systems using a 96-well plate reader. In all four sets of experiments, the concentration of each enzyme as well as substrate concentration AUR was varied with other components being held constant. It was observed that HRP (a) and AUR (c) were found to be present in excess for the concentrations tested and the GOx (b) concentration was rate-limiting under these conditions. Furthermore, when one of the enzymes was omitted, there was no generation of fluorescence which shows that all the components are necessary for the entire cascade to be triggered. There was still a slight increase in fluorescence upon removal of α-Glc (d) perhaps due to the presence of glucose impurities. Based on these bulk studies, we chose the optimal conditions for our compartment studies for sufficient product generation and detection which was 10 μM AUR, 8 U/ml GOx, 200 mU/ml HRP, 2 U/ml α-Glc, and 50 mM stachyose. Error bars are taken from the standard error of the mean (n = 3) from four independent experimental days.

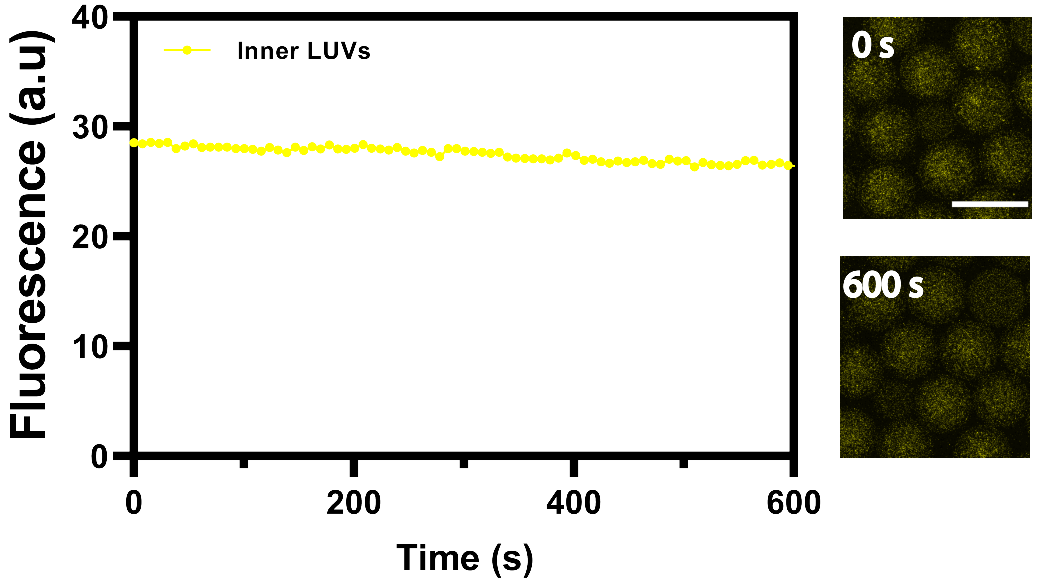

Figure S3. Intensities of encapsulated inner LUVs fluorescently labeled with Atto 390 DOPE within the two-compartment system during the reaction, together with confocal images at 0 and 600 s. Error bars are taken from the standard error of the mean (n = 40). Scale bar: 100 μm.

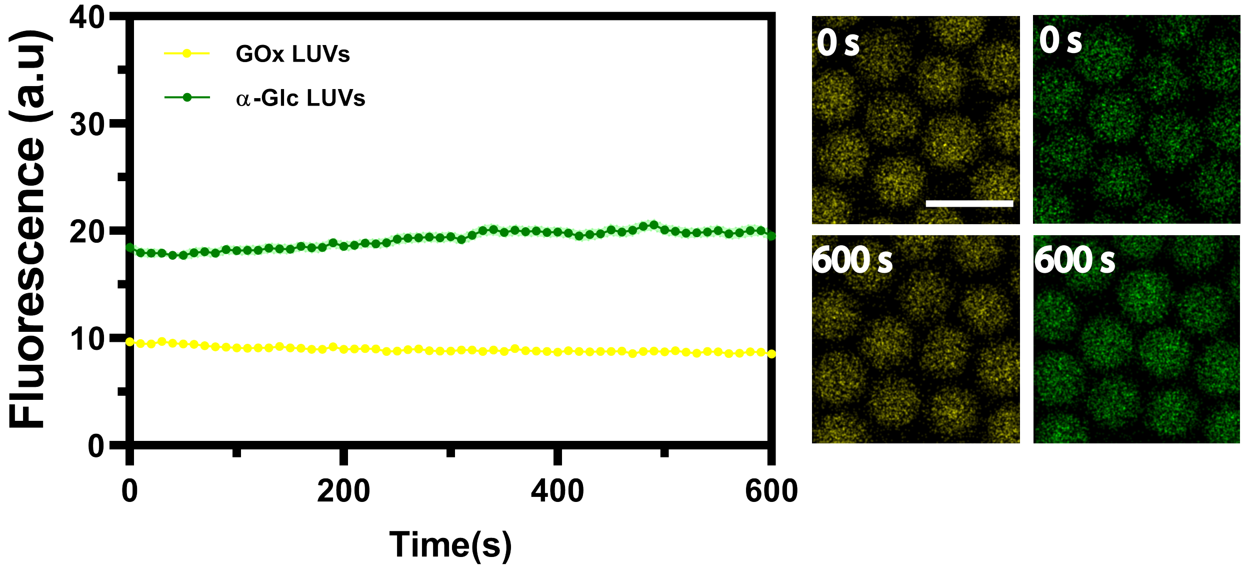

Figure S4. Intensities and confocal images of encapsulated GOx-LUVs fluorescently labeled with Atto 390 DOPE (yellow channel) and α-Glc-LUVs labeled with NBD-PE (green channel) within the three-compartment system during the reaction. Error bars are taken from the standard error of the mean (n = 30). Scale bar: 100 μm.

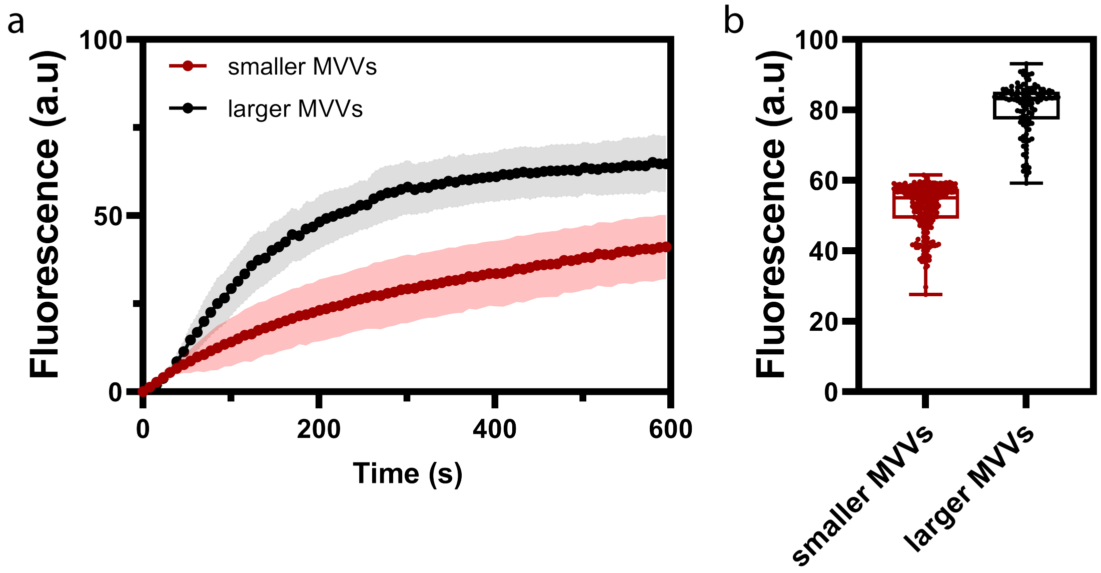

Figure S5. The effects of size on the product formation demonstrated with the three-compartment system. (a) Kinetic traces of resorufin formed in MVVs with mean diameter of 42.9 ± 4.1 μm (red) and larger MVVs with mean diameter 73.1 ± 7.2 μm (black) (b) Box plots depicting the endpoints analysis of two sizes (n ≥ 116).

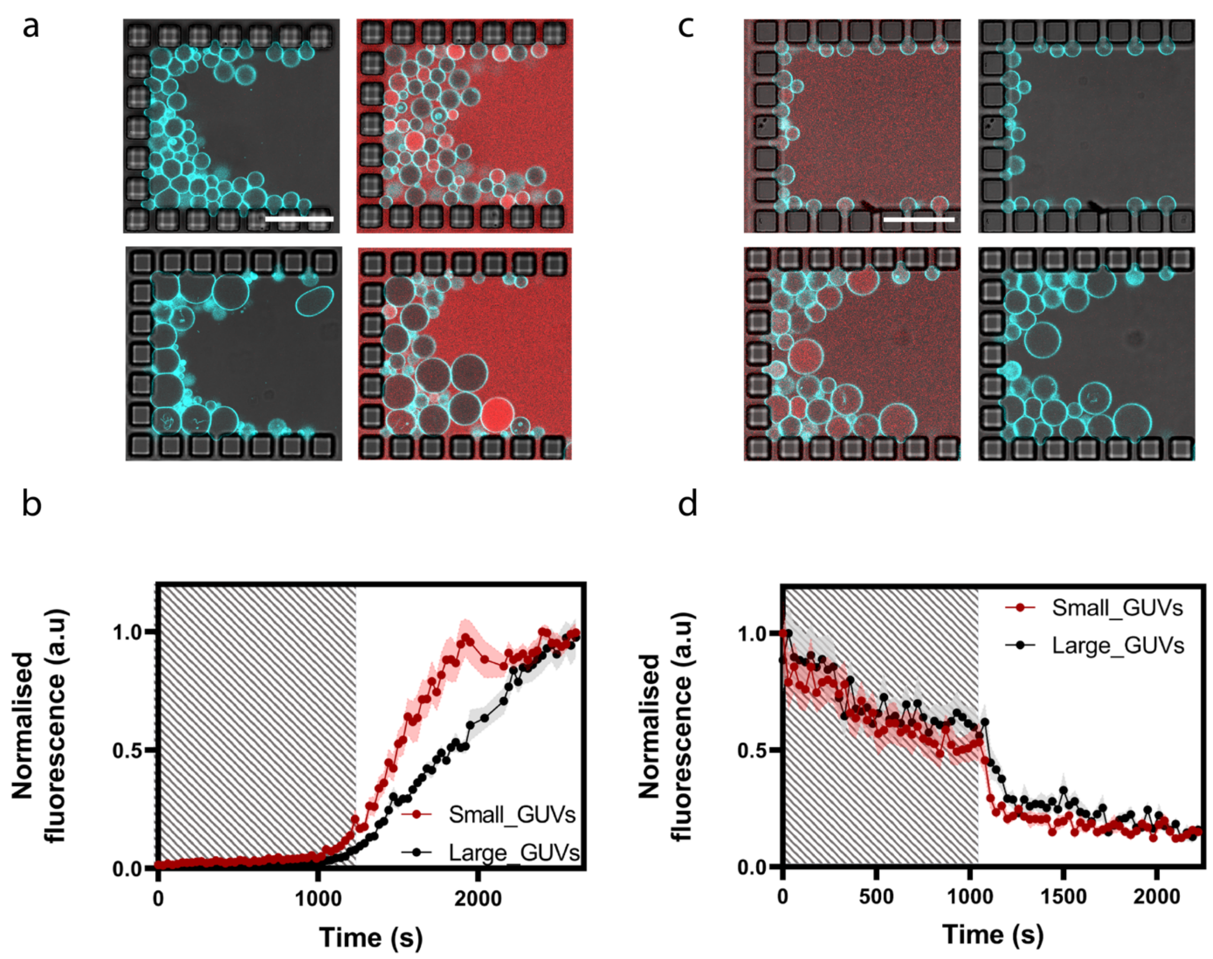

Figure S6. Diffusion of resorufin both into and out of the GUVs. (a) Confocal images of small GUVs (top panel) with a mean size diameter of 22.6 ± 2.5 μm and large GUVs (bottom panel) with a mean size diameter of 45.6 ± 3.7 μm in microfluidic traps before (left) and after (right) resorufin diffusion into their lumen. Images are overlays of fluorescent GUVs (blue) and resorufin (red) together with the trapping posts (bright-field). (b) Normalized intensities of resorufin diffusing into small GUVs (black) and large GUVs (red). Half-time (τ) values for diffusion of resorufin molecules into the lumen of GUVs were $\tau_{\text{small GUV}} =$ 1507.91 ± 14.26 s and $\tau_{\text{large GUV}} =$ 1687.73 ± 16.39 s respectively. (c) Confocal images of small GUVs (top panel) and large GUVs (bottom panel) in microfluidic traps before (left) and after (right) resorufin leakage out of their lumen. (d) Normalized intensities of resorufin diffusing out of small GUVs (black) and large GUVs (red). For the diffusion of resorufin molecules out of the lumen of GUVs, the values obtained were $\tau_{\text{small GUV}} =$ 622.83 ± 203.88 s and $\tau_{\text{large GUV}} =$ 899.46 ± 76.05 s respectively. Grey regions are the fluidic exchange times. Scale bars: 100 μm.

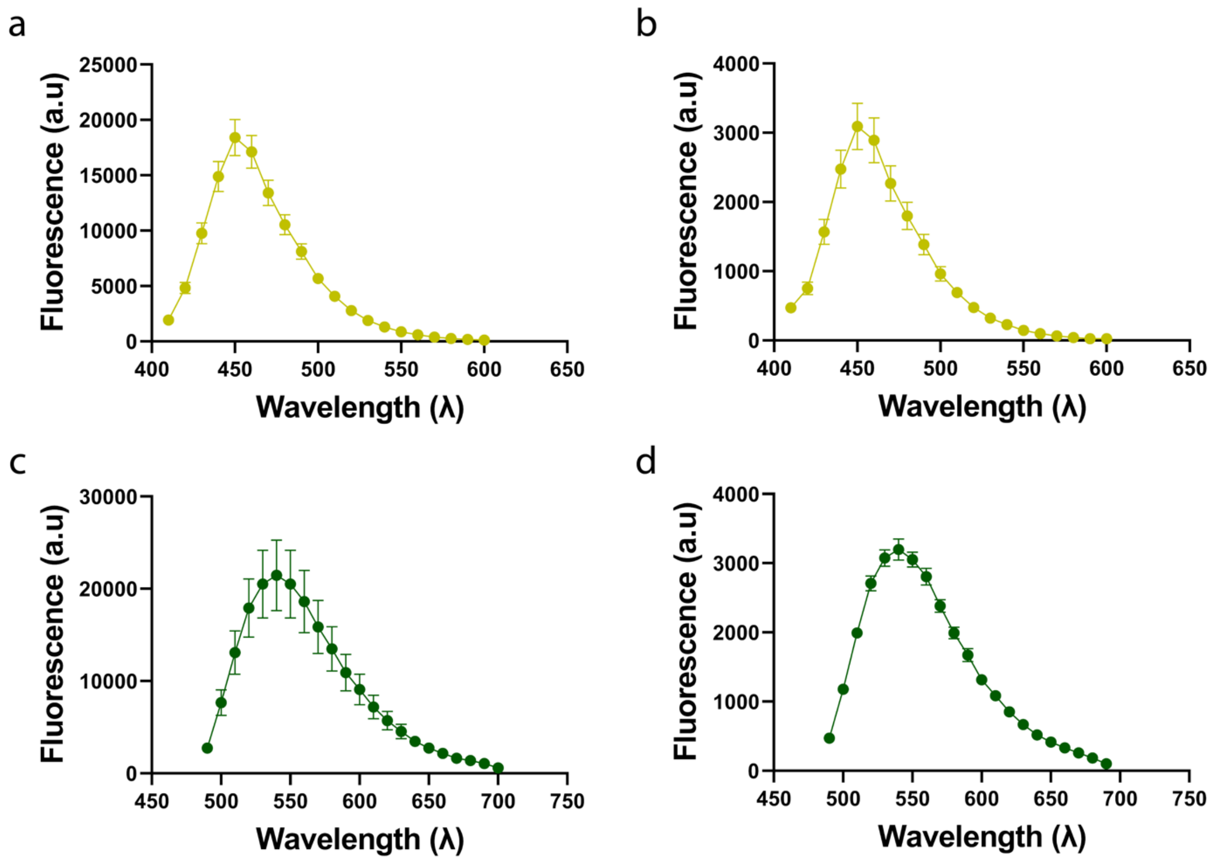

Figure S7. LUV lipid concentration calculations using a Triton-X 100 solubilization assay. Fluorescence spectra obtained after solubilization of LUVs with (a) Atto-390, (c) NBD labelled lipids with starting lipid concentration 5 mM, and (b) Atto-390 (d) NBD labelled lipids after passing through PD-10 columns. Error bars in are taken from the standard error of the mean (n = 3).

Supplementary Movie legends

Supplementary Movie 1

Microfluidic production of MVVs using a 1-inlet device with LUVs prepared in 1X PBS buffer as the IA and 1X PBS buffer solution in the OA and 5mg/mL lipid mix in 1-Octanol as the LO shown at 15 fps. Scale bar: 100 μm.

Supplementary Movie 2

Microfluidic production of MVVs using a 2-inlet device with LUVs containing two different enzymes (*i.e.* GOx and α-Glc) in 1X PBS buffer as the IA for the 2 inlets, 1X PBS buffer solution in the OA and 5 mg/mL lipid mix in 1-Octanol as the LO shown at 15 fps. Scale bar: 100 μm.

Supplementary Movie 3

Time-lapse video of the three-compartment system containing two populations of LUVs containing GOx (yellow) or α-Glc (green) encapsulated within microfluidic GUVs (magenta) over a period of 9 min. Scale bar: 100 μm.

Supplementary Movie 4

Resorufin formation (red) in the three-compartment system when triggered externally over a period of 9 min during which the signal plateaued. Scale bar: 100 μm.
